# Soluble activin type IIB receptor improves fracture healing in a closed tibial fracture mouse model

**DOI:** 10.1371/journal.pone.0180593

**Published:** 2017-07-13

**Authors:** Tero Puolakkainen, Petri Rummukainen, Jemina Lehto, Olli Ritvos, Ari Hiltunen, Anna-Marja Säämänen, Riku Kiviranta

**Affiliations:** 1 Institute of Biomedicine, University of Turku, Turku, Finland; 2 Department of Physiology, University of Helsinki, Helsinki, Finland; 3 Terveystalo Pulssi, Turku, Finland; 4 Division of Endocrinology, Turku University Hospital, Turku, Finland; Van Andel Institute, UNITED STATES

## Abstract

Fractures still present a significant burden to patients due to pain and periods of unproductivity. Numerous growth factors have been identified to regulate bone remodeling. However, to date, only the bone morphogenetic proteins (BMPs) are used to enhance fracture healing in clinical settings. Activins are pleiotropic growth factors belonging to the TGF-β superfamily. We and others have recently shown that treatment with recombinant fusion proteins of activin receptors greatly increases bone mass in different animal models by trapping activins and other ligands thus inhibiting their signaling pathways. However, their effects on fracture healing are less known. Twelve-week old male C57Bl mice were subjected to a standardized, closed tibial fracture model. Animals were divided into control and treatment groups and were administered either PBS control or a soluble activin type IIB receptor (ActRIIB-Fc) intraperitoneally once a week for a duration of two or four weeks. There were no significant differences between the groups at two weeks but we observed a significant increase in callus mineralization in ActRIIB-Fc-treated animals by microcomputed tomography imaging at four weeks. Bone volume per tissue volume was 60%, trabecular number 55% and bone mineral density 60% higher in the 4-week calluses of the ActRIIB-Fc-treated mice (p<0.05 in all). Biomechanical strength of 4-week calluses was also significantly improved by ActRIIB-Fc treatment as stiffness increased by 64% and maximum force by 45% (p<0.05) compared to the PBS-injected controls. These results demonstrate that ActRIIB-Fc treatment significantly improves healing of closed long bone fractures. Our findings support the previous reports of activin receptors increasing bone mass but also demonstrate a novel approach for using ActRIIB-Fc to enhance fracture healing.

## Introduction

Fracture healing is a complex process consisting of multiple events, which happen both simultaneously and in succession, aiming to restore the initial form and function of the fractured bone. This process is strictly regulated by numerous different cytokines, growth factors, proteases and angiogenic factors [1–3]. Recent advances in this research field have also highlighted the importance of muscle-bone cross-talk in the formation of new bone during fracture healing [4].

Numerous growth factors and signaling pathways have been identified to regulate bone remodeling. Many of these growth factors have also been shown to participate in different phases of fracture healing. Therefore, they have also emerged as potential therapeutic agents [5]. Most notable ones include members of the Transforming Growth Factor β (TGF-β) superfamily. Specific members of this superfamily, such as Bone Morphogenetic Proteins (BMPs), have shown auspicious results in different *in vivo* models in enhancing bone formation, although their efficacy has been questioned in recent clinical trials [6–9]. Furthermore, due to the lack of long-term control studies and high costs, they are yet to rival autogenous bone grafts in clinical practice, which are still conceived as the golden standard in reconstructing bone defects [10].

Activins are pleiotropic growth factors belonging to the TGF-β superfamily, which have also been linked to different pathophysiological processes [11–14]. Activin type II receptors, which initiate the activin signaling cascade consisting of activin type I and II receptors as well as receptor-regulated/common-mediator Smads, have recently been identified as novel therapeutic approaches to increase bone mass [15]. Recombinant fusion proteins of these receptors, ActRIIA-Fc and ActRIIB-Fc, function by trapping numerous ligands, including activins, and thus inhibit the ligand functions. We and others have recently shown that treatment with these activin inhibitors greatly increases bone mass systematically in different animal models [16–18]. Additionally loss of type II BMP-receptor has been reported to increase osteoblast activity and lead to increased bone mass [19].Recent report also shows that treatment with ActRIIA-Fc promotes callus formation in a closed femoral fracture model in rats [20]. Another study, however, suggested that the administration of Bimagrumab, an anti-ActRII antibody that directly inhibits the ActRII receptor instead of trapping its ligands, does not have a major impact on fracture healing [21]. Therefore further clarification of the effects of activin signaling pathways on fracture healing is needed.

We set out to investigate the effects of ActRIIB-Fc on fracture healing using a standardized, closed, diaphyseal tibial fracture mouse model. We hypothesized that inhibition of activin type IIB receptor ligands using ActRIIB-Fc would improve callus mineralization and increase bone formation resulting in accelerated fracture healing.

## Materials and methods

### Animals

In this experiment 12–14 week old male C57Bl/6 mice were used (Harlan Laboratories B.V, Netherlands). The animals were housed individually in cages under standard laboratory conditions (temperature 22°C, light from 6:00AM to 6:00PM.) Water and soy-free food pellets were available ad libitum, excluding a four-hour fasting period before euthanization. Two animals were sacrificed by CO_2_ asphyxia followed by cervical dislocation due to post-operative complications and three animals developed a severe rash behind the back of the neck during the experiment. This was observed in both PBS- and ActRIIB-Fc-treated mice and was most likely related to the C57BL/6 background of the animals. These animals were sacrificed by CO_2_ asphyxia followed by cervical dislocation and omitted from the analyses. Fractures that did not meet the standard criteria were also excluded. Altogether 70 calluses were analyzed.

### Surgical procedure

Under isoflurane anesthesia (250-400ml/min 2.5%) and aseptic surgical conditions, injections of buprenorfin (0.05mg/kg) and carprofen (5mg/kg) were administered subcutaneously. A vertical incision was made over the patellar region of the right hind leg. This was then followed by a vertical incision through the patellar tendon exposing the proximal head of the tibia. A 25-gauge needle was used to drill a hole through the cortical bone above the tibial tuberosity. A sterile Ø0.2mm stainless-steel rod was then inserted into the tibial intramedullary canal reaching the distal end of the tibia. The wound was then closed with two non-absorbable simple interrupted sutures and an anti-septic (Betadine, Takeda) was applied locally on top of the closed wounds. Standardized, closed diaphyseal tibial fractures were then performed using a fracture apparatus as previously reported [22, 23].

After the fracture was produced, the mice were closely monitored and placed on heating beds to maintain standard body temperature. Upon waking up the animals were placed in individual cages and were allowed a post-operative healing time of three to four days with administration of postoperative analgesic injections of buprenorfin and carprofen for the first two days.

### Study design

The mice were divided into two groups and were given either PBS or ActRIIB-Fc prepared in PBS (5mg/kg) intraperitoneally (i.p.) once a week. Bodyweights were recorded before every injection. Animals were sacrificed either on day 15 (two-week time point) or 29 (four-week time point) by CO2 overdose and cervical dislocation followed by sample collection.

### Production of ActRIIB-Fc

The production of the ActRIIB-Fc protein used in this study has been reported earlier [24, 25]. Briefly, the growth factor receptor consists of the ectodomain of human ActRIIB-Fc joined with IgG1-Fc. It was then expressed in Chinese hamster ovary (CHO) cells grown in a suspension culture.

### Preparation of tibia samples

The right hind legs were gathered and prepared for microcomputer tomography and histological analyses, biomechanical analyses or RNA analyses. For the first set of analyses, fracture calluses were stored in 3.8% formaldehyde for a period of 24 hours. The samples were then rinsed with PBS and stored in 70% EtOH in a dark room with a temperature of 8°C. The muscles and tendons were then removed while preserving the callus. The metal rod was removed from the proximal end of the tibia before micro computed tomography imaging (n = 6–8 per group). For the biomechanical analyses the calluses were gathered and the muscles, tendons and the intramedullary rod were removed immediately after animal euthanization. The bones were then separately wrapped in PBS-soaked gauzes and tin foil and then stored in -20°C. The samples were thawed right before biomechanical testing. (n = 5–8 per group). For the RNA analyses, fracture calluses were extracted, removed of the attached muscle soft tissue, snap frozen in liquid nitrogen and stored in -80°C. (n = 7–8 per group).

### Measurement of gene expression

For total RNA isolation, the calluses were pulverized under liquid nitrogen, homogenized in TriSure reagent (Bioline) after which RNA was extracted. The samples were treated with DNAase treatment followed by RNA clean up using RNeasy mini kit (Qiagen, Germany). The cDNA was then synthesized from 1μg of total RNA with the SensiFAST probekit (Bioline, UK). Quantitative real-time PCR was performed using iQ SYBR Green Supermix (Bio-Rad laboratories, USA). The relative mRNA expression levels were then analyzed and quantified using the 2-ΔΔCT method. The expression levels were normalized to β-actin, which was used as the internal control. Primer sequences are available upon request.

### Micro-computed tomography (μCT) analysis

X-ray micro-computed tomography of the fractured area was done using SkyScan 1070 μCT Scanner (SkyScan, Kontich, Belgium) to assess the structure of the callus using the following settings: voxel resolution of 5.33 um, X-ray potential of 70kVp, current of 200uA and an integration time of 3900ms. The object was rotated in 0.45° steps throughout the scanning for a total revolution of 182.45°. Reconstruction of the scanned images (Nrecon 1.4, Skyscan) was then done with identical settings (misalignment < 3, ring artifacts reduction 11, beam hardening correction 95%, and intensity gap 0.014–0.13). The corresponding region of interest (ROI) was drawn by the outlines of the callus (CTan 1.4.4, SkyScaN). The first and last set points of the ROI were chosen as where the fracture line was distinctly evident and the cortex of the tibial bone was not intact. The threshold values were chosen by binarization and threshold delineation so the analysis only accounted for the newly formed mineralized tissue and not the pre-existing cortical bone. Bone mineral density values were calibrated using two phantoms (calcium hydroxyapatite discs with densities of 0.25g/cm3 and 0.75g/cm3 respectively) during the scanning phase. The results were then quantified and analyzed (Batman, Skyscan).

### Histological analysis

After μCT imaging the samples were decalcified in formic acid, embedded in paraffin and then sectioned using a microtome along the sagittal plane of the middle of the callus. The sections were then deparaffinized, rehydrated and stained with hematoxylin and eosin (H&E) according to the standard protocols. Histological analyses to assess bone parameters were measured blinded for the treatment using Osteomeasure–system (Osteometrics, USA). The region of interest consisted of the callus without the periosteum, fracture ends of cortical bone or the bone marrow cavities. In the two-week samples the amount of cartilage and woven bone was analyzed and in the four-week samples the amount of trabecular bone inside the callus was assessed. Woven bone was differentiated from trabecular bone by the structure of the trabecular islands and the amount of cellularity within the trabeculae. Cartilage area was determined by the presence of prehypertrophic and hypertrophic chondrocytes based on the H&E staining.

### Immunohistochemical staining

4μm sections were cut from the paraffin-embedded samples for immunohistochemical analyses. Sections were pre-incubated with 3% bovine serum albumin (BSA) prior to the primary antibody reaction. Immunohistochemical staining was performed using the following primary antibodies: Cathepsin K (AF9210, Acris, Germany), Runx2 (D1H7 #8486, Cell Signaling Technology, USA), P-Smad1 (Ab73211, Abcam, UK) and P-Smad2 (Ab188334, Abcam, UK). The secondary antibody: Poly-HRP-Anti-Rabbit IgG (Immunologic, Netherlands). 3–3’Diaminobenzidine (DAB) was used as the chromogen and sections were counterstained with Mayer’s hematoxylin. Antibody-positive cells were then quantified blinded from a representable area (1mm^2^) inside the callus of each sample.

### Testing of biomechanical properties

A three-point-bending test was performed to evaluate biomechanical strength of the fracture calluses using a universal testing machine (Lloyd Instruments LRX, Lloyd Instruments Ltd., Fareham, Uk). The fractured tibias were placed in a lateral position and the proximal and distal ends were fixed to two in-house support pins (span = 9mm) to ensure sample stability during the procedure. The anterolateral surface of the middle part of the callus was subjected to vertical compression at a constant velocity (4.5mm/min) until breaking of the callus occurred. The measured data was converted into a load-displacement graph in real-time and the numerical values for the maximum force, stiffness and Young’s modulus of bending were then analyzed.

### Statistical analyses

All of the analyses were subjected to statistical evaluation and are shown as mean and standard deviations of the values (±). Student’s t-test as was used to assess statistical significance where the p value was set to 0.05. All statistical analyses were done with IBM SPSS Statistics v.20 (IBM, USA).

### Ethics statement

This study protocol including all the procedures was approved by the National Animal Experiment Board ELLA (license: ESAVI/11044/04.10.07/2014). All animal experiments were performed strictly according to the approved protocol.

## Results

### ActRIIB-Fc treatment augments body weight gain during fracture healing

The body mass increased significantly more in ActRIIB-Fc-treated mice compared to PBS controls during the experiment (Table 1) ActRIIB-Fc treatment induced a 12.6% increase in the body weight during the first week vs 6.6% increase in PBS groups (p<0.001) in relation to baseline and a 18.8% increase during the second week vs 8.6% increase in PBS groups (p<0.001) when compared to baseline weight (Table 1). At the third and fourth week the changes were more modest as shown in Table 1.

**Table 1:** Changes in body weight compared to the start of the experiment (%).

	1 week	2 weeks	3 weeks	4 weeks
PBS	6.6 ± 4.9	8.6 ± 5.3	10.9 ± 5.7	11.5 ± 6.3
ActRIIB-Fc	12.7 ± 3.7 ***	18.5 ± 5.2 *** **	21.5 ± 7.6 ***	24.7 ± 7.5 ***

Mean ± SD *** = p<0.001 vs PBS group

### ActRIIB-Fc robustly accelerates fracture healing and callus mineralization

**Fig 1:**
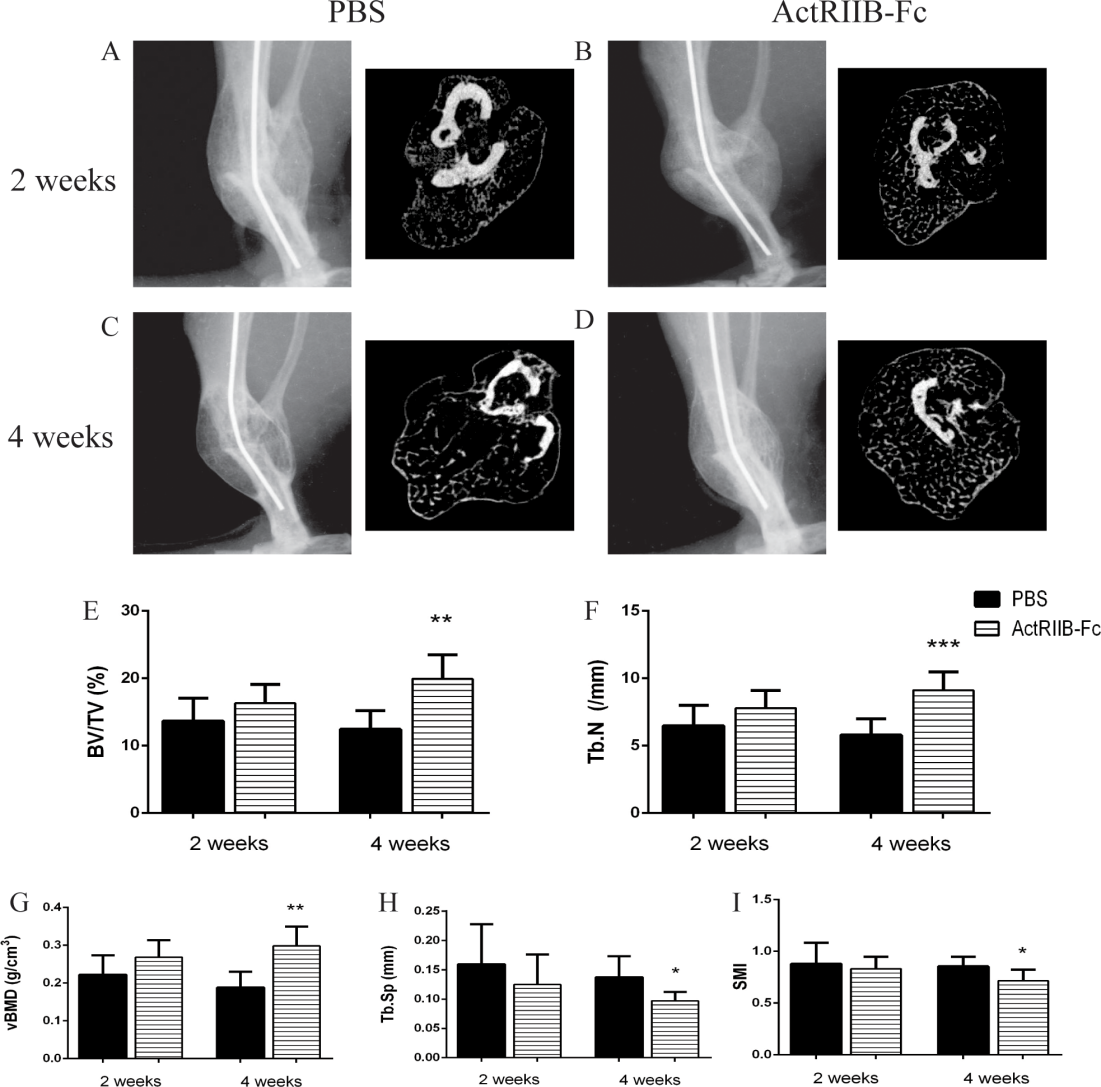
ActRIIB-Fc robustly accelerated fracture healing and callus mineralization. (A-D) Representative radiographic and micro-computed cross-sectional images of the fracture callus of the PBS- and ActRIIB-Fc-treated mice at two and four weeks. There were no significant differences in bone structure between the groups at the two-week time point. (E-I) At four weeks, ActRIIB-Fc treatment resulted in greater increases bone volume/tissue volume, trabecular numbers and volumetric bone mineral density as well as decreased trabecular separation and structural model index-* = p<0.05, ** = p<0.01, *** = p<0.001. n = 8 for PBS 2 weeks, 7 for ActRIIB-Fc 2 weeks, 8 for PBS 4 weeks and 6 for ActRIIB-Fc 4 weeks.

Radiographic images indicated no major differences between the fracture callus opacity in the two week groups but at four weeks the degree of callus maturation was greatly improved due to ActRIIB-Fc treatment (Fig 1A–1D). Cross-sectional μCT images demonstrated a greater amount of newly formed trabeculae inside the callus. A μCT analysis was done to assess the trabecular bone structure and bone mineral density in the calluses. At the two-week time point there were only minor differences between the PBS- and ActRIIB-Fc-treated groups. Slight increases in bone volume per tissue volume (BV/TV), trabecular numbers (Tb.N) and volumetric bone mineral density (vBMD) were observed in ActRIIB-Fc-treated mice but these were not statistically significant (Fig 1E–1G). However, at four weeks these differences were very prominent. ActRIIB-Fc treatment resulted in a greater bone volume per tissue volume BV/TV (+60%, p<0.01), Tb.N (+55%, p<0.001) and vBMD (+55%, p<0.01) compared to PBS control group. A clear decrease in trabecular separation (Tb.Sp) of the ActRIIB-Fc calluses was noted as well (-29%, p<0.05.) (Fig 1H) A difference in the structural model index (SMI) difference was also seen (-16%, p<0.05) in the ActRIIB-Fc-treated mice suggesting a better trabecular structure of the newly formed callus (Fig 1I).

### Treatment with ActRIIB-Fc improved the callus mechanical strength

**Fig 2:**
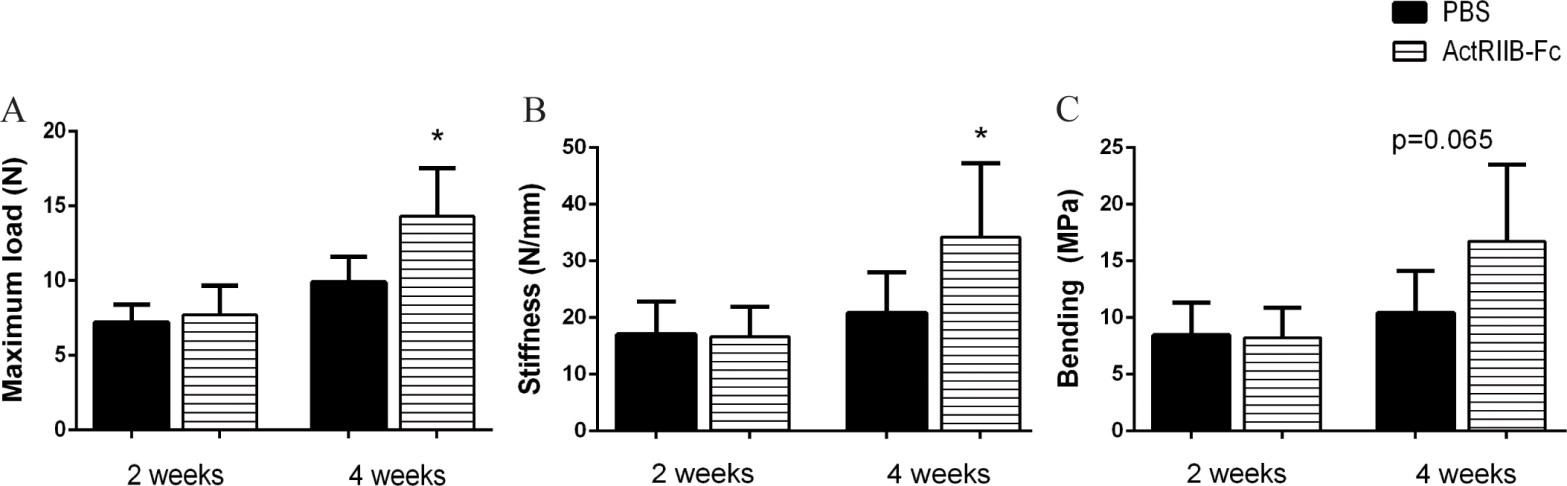
Treatment with ActRIIB-Fc improves mechanical strength of calluses. ActRIIB-Fc increases callus strength in the four week groups compared to PBS controls in terms of (A) maximum load, (B) stiffness and (C) bending strength. No significant changes were noted between the two week groups. * = p<0.05 n = 6 for PBS 2 weeks, 8 for ActRIIB-Fc 2 weeks, 5 for PBS 4 weeks and 7 for ActRIIB-Fc 4 weeks.

The three-point-bending test was used to assess the changes in biomechanical properties of the calluses between the control and treatment groups (Fig 2). There were no significant differences in the biomechanical strength of the calluses between PBS and ActRIIB-Fc groups at two weeks. At the four-week time point ActRIIB-Fc treatment resulted in improved bone strength as maximum load increased by over 45% (p<0.05) (Fig 2A) and stiffness by 65% (p<0.05) (Fig 2B). A trend in Young’s Modulus of bending was noticed as it improved by 61% (p = 0.065) (Fig 2C) compared to PBS controls. These results demonstrate that ActRIIB-Fc treatment enhances the strength of the callus compared to PBS controls.

### Histological analysis of the calluses

**Fig 3:**
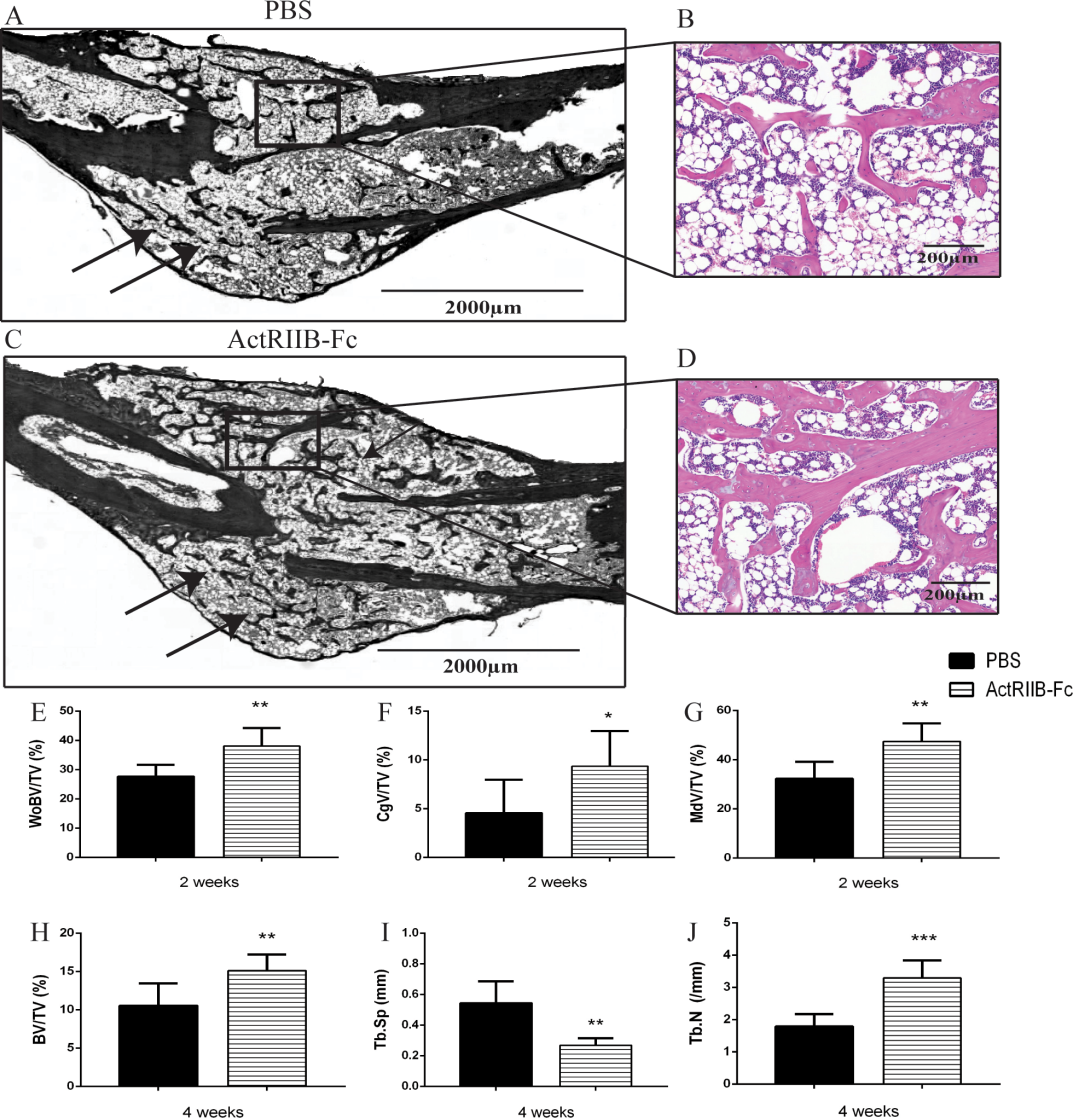
Histological analysis of the calluses. Representative hematoxylin and eosin stained histological images of fracture calluses at four weeks. (A and C) Overview image of the callus (in black and white in order to distinguish the newly formed trabecular bone more easily) and (B and D) larger magnification of the trabecular bone within the callus at four weeks. Black arrows pinpoint newly formed trabeculae. At the two week time point, ActRIIB-Fc treatment caused increased (E) woven bone volume and (F) cartilage volume compared to PBS controls resulting in increased (G) mineralized tissue per tissue volume. At the four-week time point ActRIIB-Fc treatment greatly enhanced (H) bone volume/tissue volume and (J) trabecular numbers and decreased their (I) separation * = p<0.05 ** = p<0.01, *** = p<0.001. n = 6 for PBS 2 weeks, 6 for ActRIIB-Fc 2 weeks, 7 for PBS 4 weeks and 7 for ActRIIB-Fc 4 weeks.

Histological analysis indicated significant differences in callus structure and composition in both the two- and four-week time points between ActRIIB-Fc-treated and PBS groups. At two weeks ActRIIB-Fc treatment resulted in larger amount of woven bone (WoBV/TV +38%, p<0.01) and cartilage (CgV/TV +106%, p<0.05) (Fig 3E–3G.) The combined woven bone and cartilage volume increased greatly (MdV/TV +47% p<0.01) due to ActRIIB-Fc. Similarly to the two-week time point, at four weeks ActRIIB-Fc treatment increased the amount of trabecular bone (BV/TV +44%, p<0.01 and Tb.N + 84%, p<0.001) while Tb. Sp was significantly decreased (-51%, p<0.001) (Fig 3H–3J) compared to the control group suggesting that ActRIIB-Fc treatment resulted in elevated numbers of larger trabeculae which are more densely adjacent to each other (Fig 3H–3J).

### Increased expression of osteogenic marker genes in ActRIIB-Fc-treated calluses at two weeks

**Fig 4:**
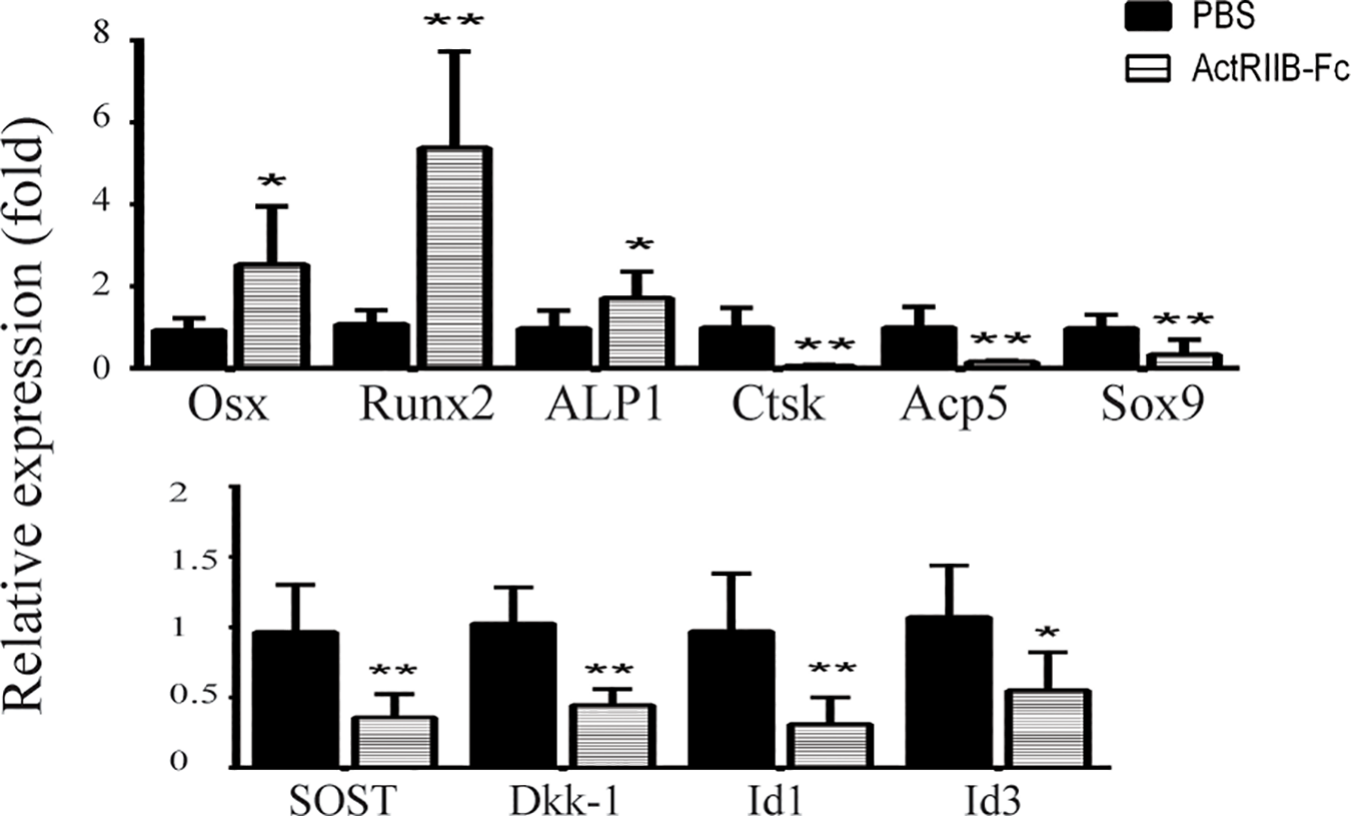
Increased expression of osteogenic marker genes in ActRIIB-Fc-treated calluses at two weeks. Quantitative real-time PCR analyses of the two-week time point revealed higher expression of essential osteoblast markers osterix, runt-related transcription factor 2 and alkaline phosphatase. Expression of cathepsin K and tartrate resistant acid phosphatase decreased compared to PBS controls which could demonstrate impaired cartilage and bone resorption. Furthermore expression of Sox9 was also lower compared to controls. Expression levels of Sclerostin and Dkk-1, negative regulators of Wnt-signaling, were also lower in ActRIIB-Fc-treated mice. Expression of Smad1/5/8 target genes Id1 and Id3 were decreased as well. * = p<0.05, ** = p<0.01. n = 7 for PBS groups and 8 for ActRIIB-Fc groups.

Quantitative real-time PCR analysis was done to assess the expression levels of specific gene markers that are known to be expressed during fracture healing (Fig 4). ActRIIB-Fc treatment resulted in 2.5-fold increase in *Osterix* (p<0.05) and 5-fold increase in *Runx2* (p<0.01) expression compared to controls. As both of these transcription factors are required for normal osteoblast differentiation, these data suggests that ActRIIB-Fc treatment enhances osteoblastogenesis and bone formation. This is further supported by the increased expression of Alkaline phosphatase (*ALP1*) expression also increased by 76% (p<0.05) compared to PBS controls. Conversely, the expression levels of markers of osteoclast activity, cathepsin K (*Ctsk*) and Tartrate resistant acid phosphatase (*ACP5*), decreased by 94% and 85%, respectively (p<0.01 in both) indicating decreased bone resorption in ActRIIB-Fc-treated calluses. The expression of Sox9, an essential transcription factor for chondrogenesis, was significantly decreased and therefore Runx2:Sox9 expression rate was higher due to ActRIIB-Fc treatment compared to PBS controls. We also noticed significant decreases in the expression of negative regulators of Wnt-signaling including sclerostin and Dkk-1 (p<0.01 in both). Additionally we measured the expression levels of known Smad-targeted transcription factors Id1 and Id3 which were also significantly down-regulated compared to PBS controls (p<0.01 in both).

### Immunohistochemical analyses of calluses

**Fig 5:**
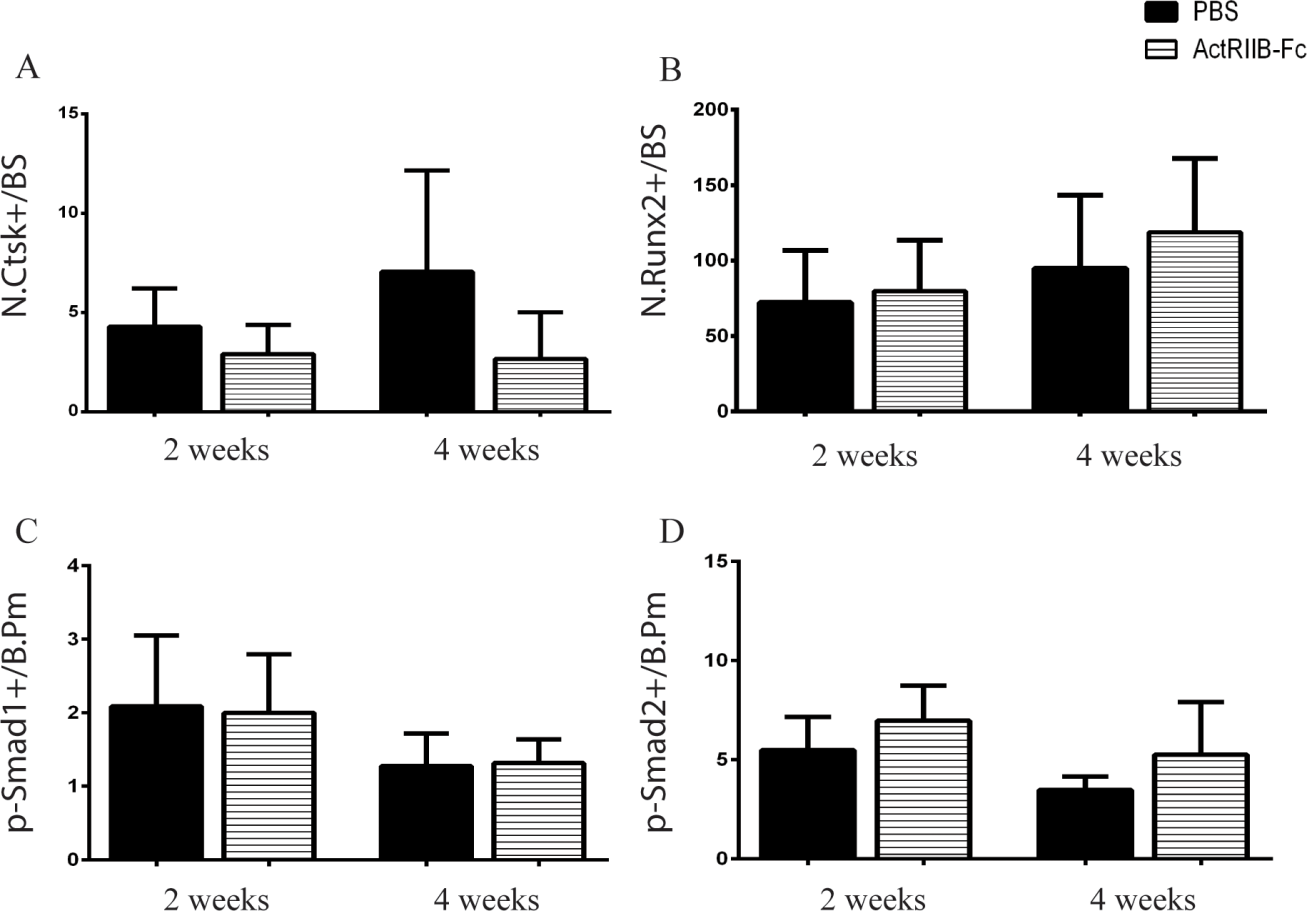
Immunohistochemical analyses of the calluses. Trends towards decreased number of cathepsin K+ cells in ActRIIB-Fc-treated mice were seen at both two and four weeks (A). The number of Runx2 positive cells was slightly increased at four weeks compared to PBS controls (p = 0.346) (B). There were no changes in the number of p-Smad1+ cells at either two or four weeks (C) but trends of increased number of p-Smad2+ cells were observed at two (p = 0.056) and four weeks (p = 0.209) due to ActRIIB-Fc-treatment (D). n = 8–9 for PBS 2 weeks, n = 10 for ActRIIB-Fc 2 weeks, n = 5–8 for PBS 4 weeks and n = 5–8 for ActRIIB-Fc 4 weeks.

To provide further evidence for increased bone formation and decreased bone resorption, we performed immunohistochemical staining on the calluses (Fig 5). Trends of decreased number of cathepsin K positive cells at both two and four weeks as well as increased number of Runx2 positive cells at four weeks were noticed and, despite being non-significant, support our results of ActRIIB-Fc enhancing callus formation. Serum levels of marker of bone resorption, C-terminal telopeptide (CTX), and marker of bone formation, N-terminal type I procollagen (P1NP), were not significantly changed at two or four weeks (S1 Fig). To elucidate the mechanism for these changes, we quantified number of phospho(p)-Smad1 and p-Smad2 positive cells in the fracture calluses. We noted no differences in the number of p-Smad1+ cells between the groups at either time point but we observed trends of increased number of p-Smad2+ cells at two and four week time points in the ActRIIB-Fc-treated mice.

## Discussion

The physiological changes associated with aging predispose older patients to musculoskeletal pathologies such as postmenopausal osteoporosis and frailty. Both of these conditions are risk factors for bone fractures. The optimal treatment approach would thus maintain or improve bone mass while enhancing/maintaining muscle mass and strength. The use of activin type II receptors as recombinant fusion proteins have already been shown to increase bone and muscle mass in different animal models but their effects on bone fracture healing are less well known. In this study we evaluated the effects of ActRIIB-Fc on fracture healing in a closed, diaphyseal tibial fracture mouse model. As we hypothesized, ActRIIB-Fc treatment improved fracture healing after four weeks of treatment when compared to PBS-treated controls. Both μCT and histological analyses at the four-week time point demonstrated increased amount trabecular bone in the calluses of ActRIIB-Fc-treated mice compared to control groups. This translated into improved biomechanical properties, as callus stiffness and strength were also increased by ActRIIB-Fc treatment. Our data indicate that these positive effects of ActRIIB-Fc were likely due to enhanced osteoblast differentiation and function and suppressed bone resorption in the fracture callus. Therefore, our results suggest that ActRIIB-Fc could be used as a novel approach to augment fracture healing.

Fracture healing is initiated by an early inflammatory phase followed by chondrogenesis and subsequent endochondral fracture healing. During the first two weeks, inflammatory and chondrogenic markers are highly expressed after which, the expression of markers of chondrogenesis dwells down and in turn the expression of osteogenic markers increases rapidly. At the two-week time point, cartilage volume was increased in ActRIIB-Fc-treated mice, while no effect was yet found in the callus mineralization despite increased expression of osteoblast marker genes. There are two possible explanations to this. Either the chondrocyte proliferation and differentiation were enhanced or the remodeling of cartilage-containing callus by endochondral healing is impaired due to ActRIIB-Fc treatment. Myostatin has been suggested to suppress chondrocyte differentiation, while some data imply that activin A could actually promote chondrogenic differentiation. Making the picture even more complex, activin A is an important regulator of inflammatory processes that are essential for proper fracture healing [26, 27]. Interestingly, at the two-week time point we already found decreased expression of chondrogenic marker mRNAs and increased expression of osteoblastic genes, despite the increased amount of cartilage in the callus. We also did find a non-significant trend towards increased number of Runx2 positive cells in ActRIIB-Fc group that likely represent pre- or mature osteoblasts at the four-week time point. This is in line with the increased osteoblastic gene expression at two weeks. Taken together our data suggest that the sum effect of the inhibition of ActRIIB-Fc ligands leads to accelerated early endochondral ossification, possibly by promoting chondrogenic and osteoblastic differentiation of mesenchymal progenitor cells at the fracture site.

As mentioned above, the increased amount of cartilage within the callus could also be due to impaired remodeling of the callus. Indeed, we did observe significantly lower expression of osteoclast marker genes *Ctsk* and *ACP5* (Fig 4) within the callus at two weeks. We also saw a non-significant trend of decreased number of cathepsin K positive cells within the calluses of ActRIIB-Fc-treated animals at both time points, that together with the decreased expression of ACP5 and Ctsk mRNAs could support slightly decreased osteoclast numbers in the ActRIIB-Fc treated animals. Activin A has been shown to have a context-dependent role as a positive regulator of osteoclast induced bone resorption [28]. Interestingly, myostatin was also recently implicated in promoting osteoclastogenesis [29]. Therefore inhibition of both myostatin and activin A with ActRIIB-Fc could lead to suppressed remodeling of the newly formed callus and in part explain the histological findings of the increased cartilage mass at the two-week time point. An interesting question is then whether inhibition of bone resorption has a negative effect on fracture healing. Bisphosphonates (BPs) are drugs that suppress osteoclastic bone resorption by inducing osteoclast apoptosis [30]. BPs are widely used in the treatment of osteoporosis, Paget’s disease and in skeletal metastases [31]. Both pre-clinical and clinical studies have been performed to assess the effects of BPs on fracture healing. Based on the available data BPs do not affect osteoblasts, inflammatory cells or other factors forming the soft or hard callus but the actual remodeling of the callus could be delayed [32]. However BPs administered early after the fracture do not delay fracture healing but could even accelerate the process [33]. Further studies are warranted to elucidate the specific effects of activin receptor ligand inhibition on the different phases of fracture healing and callus remodeling.

We were able to show that ActRIIB-Fc enhances fracture healing at the four-week time point with robust increases in the trabecular bone volume and bone mineral density within the callus. These changes were also translated into enhanced biomechanical properties as ActRIIB-Fc-treated calluses were stronger and stiffer compared to their PBS-treated controls. In addition, our data provide a possible mechanism for these effects as ActRIIB-Fc greatly increased the expression of osteoblast markers genes and favors the commitment of precursor cells to the osteogenic lineage over the chondrogenic one due to the increased Runx2:Sox9 expression ratio as described before [34]. This early switch from chondrogenic to osteogenic program could lead to the robust increase in mineralized bone at four-week time point. To further explore the mechanisms by which ActRIIB-Fc enhances fracture healing, we examined the activation of the BMP and TGF-β signaling pathways by analyzing the number of p-Smad1 and p-Smad2 cells within the fracture callus using immunohistochemistry (IHC). Unfortunately, IHC does not allow for evaluation of the level of signaling activity within the positive cells. Although non-significant, there was a trend towards increased number of p-Smad2 cells in the callus at both two and four weeks, which is somewhat surprising as with ActRIIB-Fc treatment one could expect decreased Smad2 activation. However, as TGF-β has been shown to recruit osteoblast progenitors to the sites of active bone remodeling [35], the increased number of p-Smad2 positive cells could be due to enhanced recruitment of early osteoblast progenitors at the fracture site. We also measured the expression of BMP signaling target genes Id1 and Id3 and both mRNAs were expressed at lower level in ActRIIB-Fc-treated calluses. The expression of Id1/3 are induced by acute BMP treatment but their expression normalizes within a few days [36]. In our experiment the animals have been treated for several weeks before mRNA analyses, possibly explaining the suppressed Id1/3 mRNA levels. Moreover, Id proteins induce early proliferation of osteoblast progenitors but prolonged Id expression appears to inhibit osteoblast differentiation. Two- and four-week time points represent phases of rapid bone formation and later remodeling i.e. high rate of osteoblast differentiation. We believe that actually the low Id expression in ActRIIB-Fc-treated animals reflects the enhanced osteoblast differentiation at these time points. Lastly, we found that the expression levels of Wnt1 signaling inhibitors Sclerostin and Dkk-1 were decreased upon ActRIIB-Fc-treatment, possibly leading to enhanced Wnt signaling. Thus ActRIIB-Fc treatment appears to induce multiple pathways to stimulate fracture healing.

Our findings are partly in concordance with the report from Morse et al. where the authors stated that ActRIIA-Fc treatment augmented callus formation in rats [20]. However, they did not find significant changes in callus BV/TV compared to the vehicle controls and the treatment with ActRIIA-Fc only modestly improved the biomechanical properties. Although the models used (rat vs. mouse, femur vs. tibia) are different to our study, these findings suggest a difference between the effects of ActRIIA-Fc and ActRIIB-Fc on fracture healing. Tanko et al. [21] in turn reported contradicting results stating that the use of intravenous bimagrumab, an anti-ActRII antibody, had no significant effects on rat fibula osteotomy healing as they observed no differences in mature callus size, vBMD or biomechanical properties and they suggested that ligands that signal through other receptors, such as specific bone morphogenetic proteins, have more relevant roles in fracture healing. However, Nagamine et al. previously demonstrated in an immunohistochemical study that ActRIIA/B are strongly expressed in mature and hypertrophic chondrocytes as well as in osteoblasts during the different phases of fracture healing suggesting that activin type II receptor ligands are important regulators of these events [37]. The differences between these and our studies could in part be explained by the differences in the treatment approaches as bimagrumab directly inhibits the ActRII receptors while our ActRIIB-Fc inhibits the binding of all of its ligands to ActRIIB as well as their alternative receptors. Moreover, the fracture models (closed and open fractures), the duration of healing and the animal species used varied between the studies.

Despite recent advances in the treatment of osteoporosis, osteoporotic fractures will remain as a significant disease burden to our society. Fractures lead to periods of immobilization, which result in further bone loss as well as loss of muscle mass that may further impair patient mobility. Moreover, fractures cause increased mortality [38]. We and others have previously shown that treatment with ActRIIB-Fc results in robust increases in both muscle and bone mass and could thus provide an intriguing treatment option for frail, osteoporotic patients [17, 39]. Here we demonstrate that in a closed tibial fracture mouse model treatment with ActRIIB-Fc results in enhanced fracture healing seen in improved callus bone volume and structure, which translated into biomechanically stronger calluses four weeks after the fracture. Our data suggests that this is likely due to increased osteoblastic bone formation and suppressed bone resorption. These data demonstrate that ActRIIB ligands play an important role in regulating multiple phases of fracture healing. Moreover, ActRIIB-Fc with its effects on bone and muscle could provide a novel approach to enhance fracture healing.

## Supporting information

S1 Fig ActRIIB-Fc treatment does not affect CTX or P1NP serum levels at two or four weeks. ActRIIB-Fc treatment does not affect CTX or P1NP levels at two or four weeks compared to PBS controls. n = 7 for all groups.
(DOCX)

S1 File Master data file. Specific values of each sample for each analysis listed as they appear in the manuscript.
(XLSX)
