# Supplementary material for: Soluble activin type IIB receptor improves fracture healing in a closed tibial fracture mouse model
Source: PLoS One. 2017 Jul 13;12(7):e0180593. doi: 10.1371/journal.pone.0180593 (PMC5509431; doi:10.1371/journal.pone.0180593)

**Soluble activin type IIB receptor improves fracture healing**

**in a closed tibial fracture mouse model**

Tero Puolakkainen, Petri Rummukainen, Jemina Lehto, Olli Ritvos, Ari Hiltunen, Anna-Marja Säämänen, Riku Kiviranta

Supporting information

**Supporting Materials and Methods**

**Serum analysis**

Exsanguination was performed and the samples were centrifuged to differentiate the serum from the blood clot. Serum samples were then collected and stored in -80°C. Serum levels of C-terminal telopeptide (CTX) and N-terminal type I procollagen (P1NP) were analyzed (ValiRx labrotarory, Finland) with RatLaps (CTX-1) EIA and Rat/Mouse P1NP EIA assay kits, respectively. Calibrators and controls were used to ensure the validity of the kits.

**Supporting Results**

**S1 Fig: ActRIIB-Fc treatment does not affect CTX or P1NP serum levels at two or four weeks.**


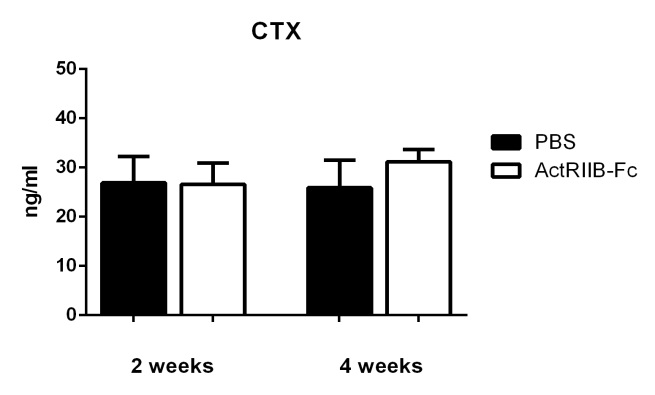

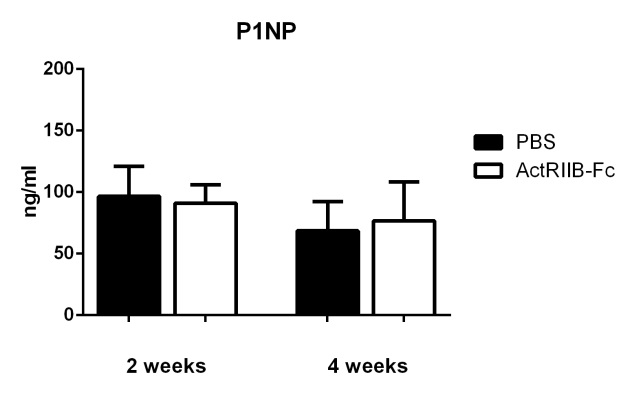

Supplement: S1 Fig — ActRIIB-Fc treatment does not affect CTX or P1NP levels at two or four weeks compared to PBS controls. n = 7 for all groups. (DOCX) [file pone.0180593.s001.docx]
